# AI Tools for Heart Failure Management: A Comprehensive Review of Potential, Pitfalls, and Predictive Analytics

**DOI:** 10.7759/cureus.94920

**Published:** 2025-10-19

**Authors:** Akshita Bhandari, Ibrahim Riaz, Sourav Hariram, Cesar R Izaguirre Vallejos, Sonali Gupta, Kavya Sharma, Elizabeth c Palaparthi, Pyae Phyo Kyaw, Charam M Reddy Seelam, Shenon Tiwary, Pavankumar Narayanan, Momin Imtiaz, Ramsha Ali

**Affiliations:** 1 Department of Internal Medicine, Adesh Institute of Medical Sciences and Research, Bathinda, IND; 2 Department of Acute Medicine, Basildon University Hospital, Basildon, GBR; 3 Department of Acute Medicine, Global Business School for Health, University College London, London, GBR; 4 Department of General Medicine, North West Anglia NHS Foundation Trust, Peterborough, GBR; 5 Department of Internal Medicine, Franz Tamayo University, La Paz, BOL; 6 Department of Cardiology, Birmingham Heartlands Hospital, University Hospitals Birmingham NHS Foundation Trust, Birmingham, GBR; 7 Department of Medicine, MM Medical College & Hospital, Kumarhatti-Solan, Solan, IND; 8 Department of Internal Medicine, Shasta Regional Medical Center, Redding, USA; 9 Department of Gastroenterology and Hepatology, Birmingham Heartlands Hospital, University Hospitals Birmingham NHS Foundation Trust, Birmingham, GBR; 10 Department of Internal Medicine, Kasturba Medical College, Manipal, IND; 11 Department of Internal Medicine, KPC Medical College & Hospital, Kolkata, IND; 12 Department of Acute Medicine, Southend University Hospital, Mid and South Essex NHS Foundation Trust, Southend-on-Sea, GBR; 13 Department of Internal Medicine, Istanbul Medipol University International School of Medicine, Istanbul, TUR; 14 Department of Medicine and Surgery, Peoples University of Medical and Health Sciences, Nawabshah, PAK

**Keywords:** artificial intelligence, cardiac mri, deep learning, driven models, echocardiography, heart failure

## Abstract

Heart failure (HF), as a sequela of cardiac insult, has long been recognized for the excessive burden it places on healthcare systems worldwide. Advancements have been made in both the interventional and pharmacological landscapes related to the disease, with monumental strides achieved in reducing morbidity and mortality. However, patients continue to live in fear of the disease as they face the risk of repeated hospitalizations, adverse outcomes, and the financial strain it imposes. Despite the vast amount of literature available to clinicians, bridging the gap between theoretical knowledge and clinical practice remains challenging due to persistent knowledge gaps. Integrating clinical data, identifying patterns in key investigations, and making informed clinical decisions are difficult, particularly when tailoring treatments to each patient’s unique characteristics. AI has shown great potential in addressing these challenges and assisting clinicians. Through this review, we aim to demonstrate how AI algorithms and models, such as machine learning, deep learning, and natural language processing, can support various aspects of HF management. This narrative review was conducted through a comprehensive and structured literature search on PubMed. Screening identified 163 articles that met the inclusion criteria from an initial total of 1,617. Data extraction included author name, study type, digital object identifier, study objective, sample size, key findings, and relevance to AI applications in HF management. Recent literature on AI and HF highlights the significant impact of AI on expanding the scope of practice in this field. Several key findings stand out: (1) AI has enhanced the detection of subclinical HF (i.e., the presence of HF without noticeable symptoms); (2) AI algorithms, when compared to traditional methods, demonstrate greater accuracy in identifying the most suitable treatment for HF according to patient characteristics; and (3) human-machine collaborative models have proven superior in predicting one-year readmission rates for patients with HF. Several challenges, such as algorithmic bias, data security concerns, the “black box” nature of AI, and other risks of bias, have also been identified. Nevertheless, with ethical oversight and regular clinical engagement, AI continues to demonstrate significant potential in HF management. With the latest advances, AI is poised to play an even greater role in transforming HF care, shifting it toward more proactive and data-driven models.

## Introduction and background

Heart failure (HF) is a leading global health burden, affecting approximately 64 million people worldwide [1]. In the United States alone, its prevalence is expected to increase by 46% by 2030, reaching 6.2 million cases [2]. Europe also bears a significant burden, with 15 million HF cases reported annually, including three million HF-related hospitalizations [2]. The economic impact of HF in the United States exceeds $30 billion, primarily due to long-term management and recurrent hospitalizations [2]. Despite advancements in pharmacotherapy and device-based therapy, HF outcomes remain suboptimal, with persistently high rates of hospitalization and mortality [3]. Nearly 25% of HF patients are rehospitalized within one month of discharge, and in recent years, Medicare beneficiaries have shown only a mild reduction in readmission rates [4]. HF drastically reduces patients’ quality of life, causing symptoms such as fatigue and poor exercise tolerance, which further increase hospitalization rates and contribute to decreased life expectancy.

Given these challenges, it is crucial to identify new strategies to improve HF management, particularly in risk stratification, early disease detection, and individualized therapeutic interventions. AI has emerged as a transformative tool in cardiovascular medicine. Using machine learning (ML) and deep learning (DL) techniques, AI can analyze large and complex datasets with high precision [5,6]. AI-powered tools can detect subtle patterns in patient data that are often missed by conventional statistical methods, thereby helping clinicians make more accurate predictions and treatment decisions [7].

For instance, DL algorithms trained on ECG data have demonstrated the ability to predict HF-related events and mortality with accuracy surpassing that of traditional risk scores [8]. Compared with conventional regression-based models, AI-based predictive models using electronic health records (EHRs) and echocardiographic imaging have shown greater efficiency in predicting hospital readmission and mortality [9]. These developments have shifted HF management from a reactive approach to a proactive one, enabling earlier interventions and more personalized treatment strategies [10].

AI applications extend beyond HF to other cardiovascular conditions, including myocardial infarction, arrhythmias, and valvular heart diseases [11]. AI-assisted diagnostic tools, such as cardiac angiography, echocardiography, and ECG analysis, have demonstrated high accuracy in disease detection and prognosis [11]. For example, convolutional neural networks (CNNs) have been used to develop ECG-based AI models capable of detecting cardiac dysfunction, estimating prognosis, and predicting clinical decline [12]. Furthermore, AI-assisted evaluation of EHRs enables stratification of HF patients according to left ventricular ejection fraction (LVEF) subtypes, thereby improving risk prediction and supporting personalized treatment planning [9,13].

Beyond hospital settings, AI has transformed patient care through remote monitoring with wearable devices. These technologies continuously track physiological parameters and vital signs, facilitating early detection of HF deterioration and enabling timely interventions when needed [14,15]. AI also plays a crucial role in managing asymptomatic conditions, such as left ventricular dysfunction, by enabling early prediction of HF exacerbations, mortality, and recurrent hospital readmissions.

Despite its promise, several obstacles hinder the widespread clinical implementation of AI in HF management. Major challenges include the interpretability of AI-generated predictions (the “black box” issue), biases in training datasets that affect model generalizability, and regulatory barriers to clinical adoption [16]. Additionally, inconsistencies in data quality and the lack of standardized reporting methods pose significant challenges to reproducing results reliably and validating AI models across diverse clinical settings [17]. Recognizing and addressing these challenges is essential to ensure the safe, effective, and equitable integration of AI-driven tools into routine clinical practice.

**Figure 1:**
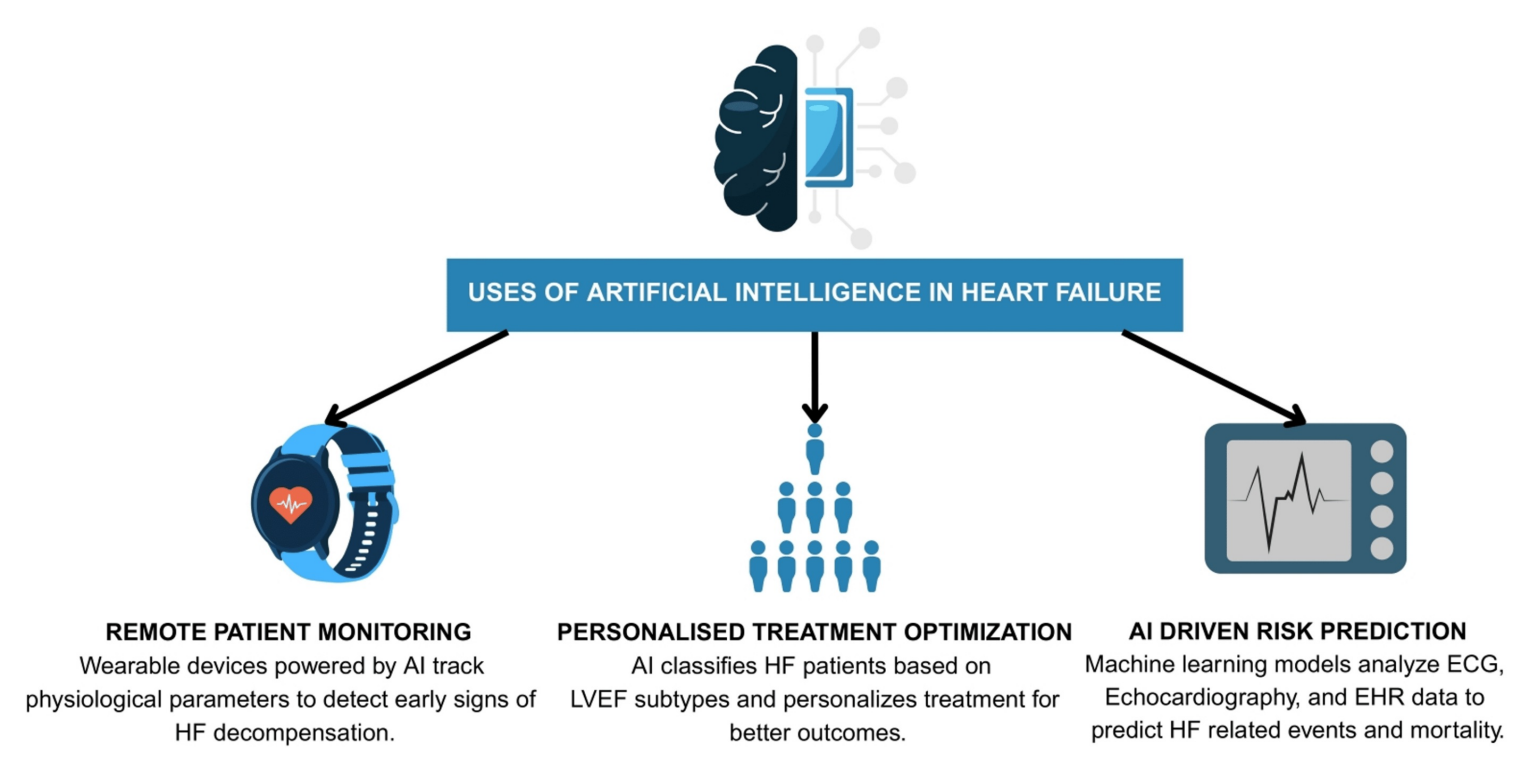
Uses of AI in HF management The illustration above highlights key applications of AI in HF management. It depicts remote patient monitoring using wearable devices, improvements in treatment personalization based on LVEF subtypes, and AI-driven risk prediction. Collectively, these approaches enhance early detection, treatment precision, and outcome prediction in HF care. EHR, electronic health record; HF, heart failure; LVEF, left ventricular ejection fraction Figure created from data from references [8,9,11-15].

This review critically examines the role of AI in HF diagnosis and management, with a focus on its potential to enhance risk prediction, therapeutic strategies, and clinical outcomes (Figure 1). By synthesizing current evidence, we aim to assess the efficacy of AI applications, identify existing gaps and limitations, and provide evidence-based recommendations for integrating AI into HF care.

## Review

Methods

This narrative review was conducted in accordance with the Preferred Reporting Items for Systematic reviews and Meta-Analyses (PRISMA) guidelines [18]. A systematic literature search was performed to identify relevant studies on the application of AI in HF management. A comprehensive and structured search was carried out on PubMed on February 12, 2025, using Medical Subject Headings (MeSH), the controlled vocabulary thesaurus of the National Library of Medicine, to ensure precision in retrieval. The search terms included “artificial intelligence”, “HF”, “management”, and “treatment”, following the Peer Review of Electronic Search Strategies (PRESS) guidelines [19]. Boolean operators (AND, OR) were applied, with the “explode” function enabled to capture all relevant subcategories. Filters were applied to limit the search to human studies, English-language publications, and articles published from 2015 onwards.

Following the initial search, the strategy was refined by reviewing titles, abstracts, and subject terms to identify any missing keywords. Two independent reviewers (SH and CRIZ) manually screened the search results against predefined inclusion criteria. Titles and abstracts were initially assessed, and full-text articles were retrieved for further evaluation if eligibility remained uncertain. Discrepancies were resolved through discussion.

Studies were included if they met the following criteria: (1) investigated the application of AI in HF management, including diagnostic assessment, prognostication, risk stratification, or therapeutic guidance; (2) reported on AI-driven predictive models using structured clinical data, imaging modalities, wearable technology, or multi-omics approaches; and (3) were peer-reviewed and published between 2015 and 2025. Studies were excluded if they (1) did not specifically examine AI applications in HF management; (2) were nonhuman studies, commentaries, or non-peer-reviewed sources; or (3) focused solely on AI model development without consideration of clinical relevance.

Following screening, 163 studies met the inclusion criteria and were included in the qualitative synthesis. Data extraction was performed by all authors using a structured framework to ensure consistency. Extracted parameters included author name, study type, digital object identifier, study objective, sample size, key findings, and relevance to AI applications in HF management. All data were compiled in a standardized Microsoft Excel spreadsheet.

Key Findings

Our narrative literature review initially identified 1,617 publications, from which 163 articles met the inclusion criteria. The selected literature encompasses a methodologically diverse array of research, including eight randomized controlled trials, four systematic reviews, one meta-analysis, four narrative reviews, and five literature reviews. The collection also includes observational studies, predictive analyses, experimental studies, post-hoc analyses, qualitative research, and pilot investigations. Based on our analysis, we identified several key themes (Table 1).

**Table 1:** Key findings: AI in HF diagnosis and management AS/ML, artificial super-intelligence/machine learning; DL, deep learning; EHR, electronic health records; HF, heart failure; LVEF, left ventricular ejection fraction; LV dysfunction, left ventricular dysfunction; ML, machine learning; SCD, sudden cardiac death; SHAP, SHapley Additive exPlanations Table created by the authors using data from references [12,20-29].

Category	Key findings	Supporting evidence
AI-guided risk identification and analysis	ML-based predictions using ECG data, biomarkers, phenotypes, and EHRs have improved risk stratification and contributed to better patient outcomes.	Analysis and prediction using AI-guided ECG and biomarkers [20]; prediction using ensemble models and interpretability tools [21]; biomarkers for risk stratification [22]
AI-powered decision support for diagnosis and treatment in HF	AI-driven analysis of patient data, imaging tools including ECG, and biomarkers facilitates early disease detection and personalized treatment plans. This technology enhances prediction of treatment responses and enables targeted therapies, resulting in improved patient outcomes.	Potential of AI in diagnosis and management of HF [23]; application of biomarkers for clinical diagnosis of HF [24]; integration of ML models into HF management [25]
ML/DL-based morbidity, mortality, and hospital admission prediction	Integrating clinical factors such as symptoms, family history, and LVEF with AI-driven models significantly improves predictions related to mortality, hospital readmissions, and sudden cardiac death.	ML-based mortality and readmission prediction [26]; ECG-based prediction of SCD in HF patients using AI [12]; predictive modeling using SHAP for hospital readmission in HF [27]; AI-guided ECG to predict LV dysfunction and prognosis in HF patients [28]
Innovation and future directions in AI-based HF care	Integrating AI with clinical and imaging data supports enhanced prediction of mortality, hospital readmissions, and SCD, while paving the way for future advancements such as AI/ML-driven myocardial recovery modeling.	Future advancements in predictive modeling using AI/ML for myocardial recovery in HF [29]

Discussion

**Figure 2:**
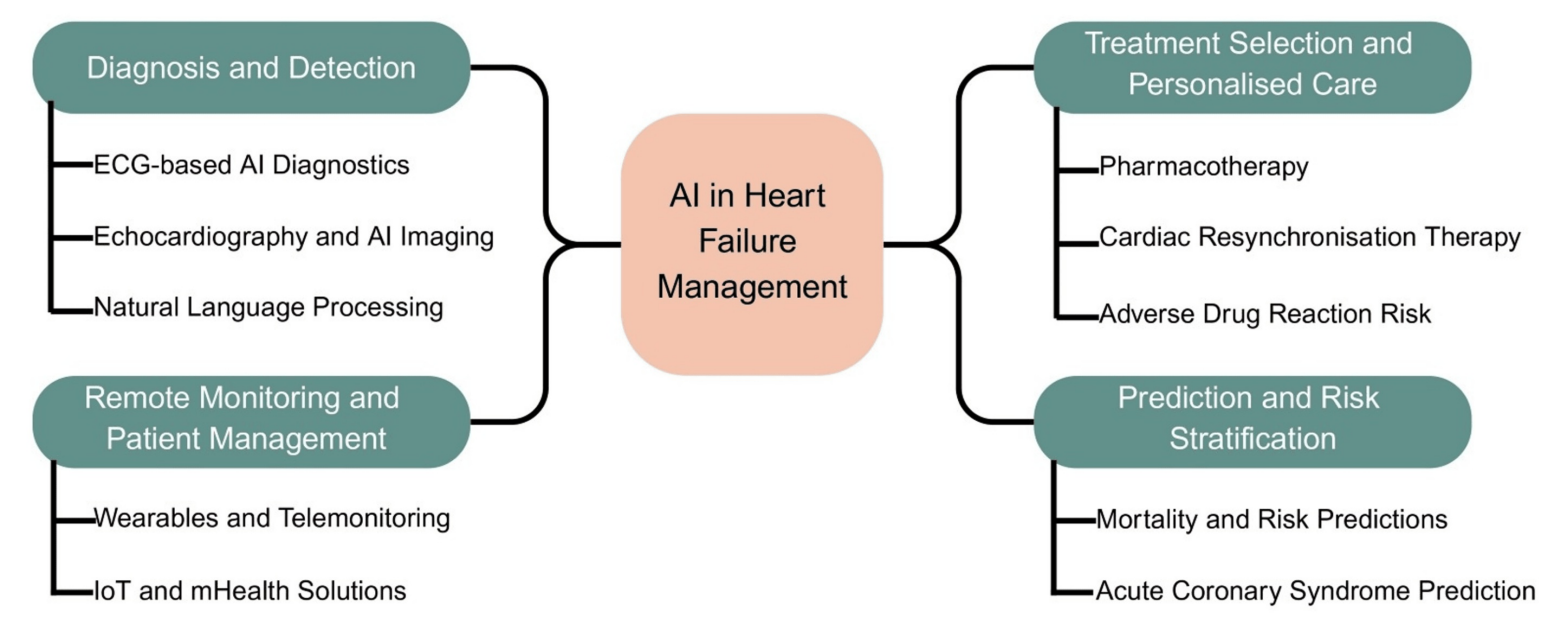
AI applications in HF management The figure illustrates key applications of AI in HF management, including diagnosis and detection using ECG-based AI diagnostics, echocardiography and AI imaging, and NLP; remote monitoring and patient management via wearable devices and telemonitoring; treatment selection and personalized care; and prediction and risk stratification, such as mortality risk and acute coronary syndrome prediction. HF, heart failure; IoT, Internet of Things; NLP, natural language processing Figure created by the authors using data from references [2-4,8] and generated with Napkin AI.

AI is revolutionizing echocardiography and cardiac MRI by automating image analysis, improving diagnostic accuracy, and potentially enhancing personalized patient care. AI algorithms can perform tasks such as image segmentation, measurement extraction, and disease detection, freeing clinicians to focus on more complex tasks and improving diagnostic consistency. In echocardiography, applications include automated segmentation and volumetric analysis of the cardiac chambers, ejection fraction (EF) calculation, assessment of valvular structures (including valve geometry and flow gradients), and measurement of longitudinal strain and cardiac wall motion abnormalities. Examples of clinical use include automated diagnosis of myocardial infarction, differentiation of hypertrophic cardiomyopathy from physiological hypertrophy, and detection of HF and pulmonary artery hypertension (PAH). When combined with handheld echocardiography, these AI applications can provide high-quality cardiac diagnoses in regions lacking such capabilities, thereby helping to democratize access to expert cardiac evaluation (Figure 2) [30].

Cardiac CT, including CT angiography, is another modality increasingly utilizing AI. Applications include automated quantification of coronary artery plaques and blood flow, as well as cardiovascular risk assessment using coronary artery calcium scoring. AI can assist in detecting various cardiac conditions, including valvular heart disease, congenital heart defects, and HF, through echocardiographic image analysis. AI can evaluate the suitability of the aortic annulus for transcatheter aortic valve replacement procedures and analyze late gadolinium enhancement images to identify myocardial scarring and fibrosis, which are key markers of heart disease. By integrating cardiac MRI data with other clinical, genetic, and omics data, AI provides a more comprehensive understanding of patient conditions. Furthermore, AI can optimize workflows in echocardiography laboratories by automating tasks and streamlining reporting [30].

Challenges of AI Implementation in Healthcare

Primary challenges of system integration include the complexity of combining disparate systems with different interfaces and technologies, ensuring data consistency and integrity across the ecosystem, managing costs and resources for integration projects, and maintaining security and regulatory compliance. Ethical issues, such as data privacy and bias, are among the most significant challenges in healthcare AI implementation. Protecting patient data and avoiding the perpetuation of social biases is essential for responsible and equitable use of AI [31].

AI regulation in healthcare is rapidly evolving, focusing on patient safety, ethical considerations, and responsible innovation. Existing regulations, such as those for Software as a Medical Device (SaMD), are being adapted to address AI-specific challenges, including its ability to learn and adapt over time. General regulations address broad AI concerns, while targeted regulations focus on specific healthcare applications. Key regulatory challenges include rapid technological advancements, model autonomy and adaptability, liability and accountability, and the need for global harmonization [31]. Current regulatory initiatives include the FDA’s AI/ML-based SaMD Action Plan, which focuses on regulating AI/ML-based software as medical devices; WHO’s Regulatory Considerations, emphasizing safety, effectiveness, and accessibility; the US-EU Trade and Technology Council’s development of a voluntary AI code of conduct; and Virginia Law (HB2154), which addresses the use of intelligent personal assistants in healthcare facilities [31].

Types of AI Modalities and Their Application in Healthcare 

Effective use of AI in healthcare requires understanding the different AI tools, each with specific functionalities and limitations. Within diagnostic imaging, ML, particularly DL, has emerged as a fundamental tool for image recognition, demonstrating significant efficacy in radiology and pathology [32]. Natural language processing (NLP) allows screening of large volumes of unstructured data, such as physician notes and EHRs, transforming it into meaningful information and enabling advanced clinical decision support systems [33,34]. ML tools also aid proactive patient management by predicting outcomes and identifying high-risk cohorts [35,36].

Application of these modalities presents unique opportunities and challenges depending on the medical condition. For example, DL in echocardiography and cardiac MRI may enhance assessment precision, while NLP can extract relevant clinical features from patient notes. However, the inherent heterogeneity of human physiology and disease poses significant challenges to developing robust and generalizable AI models.

NLP has become a valuable tool in healthcare for extracting insights from clinical texts such as EHRs, progress notes, and discharge summaries. However, its effectiveness in clinical settings is constrained by several key limitations, particularly in handling ambiguous terminology and stylistic variations in documentation. One significant challenge is ambiguous medical terminology. Many clinical terms are context-dependent; for example, the words “cold” or “MS” have different meanings depending on context. NLP systems often struggle to disambiguate such terms, which can lead to misinterpretation and reduced accuracy in clinical decision support or data extraction. Another limitation is variability in clinical documentation styles across institutions, departments, and individual clinicians. These stylistic differences pose challenges for NLP models in accurately interpreting and extracting relevant information [37].

Mitigation strategies to reduce these errors include the use of domain-specific ontologies such as SNOMED CT or UMLS, which provide standardized mappings for ambiguous terms. Context-aware NLP models, such as transformer-based architectures (e.g., BioBERT and ClinicalBERT), can disambiguate terms by learning from broader textual context. Additionally, incorporating human-in-the-loop systems, where clinicians validate or correct NLP outputs, can enhance model reliability. Hence, while NLP in clinical contexts faces limitations due to ambiguity and variability, careful model design, domain knowledge integration, and continuous validation can significantly mitigate these challenges [38].

**Figure 3:**
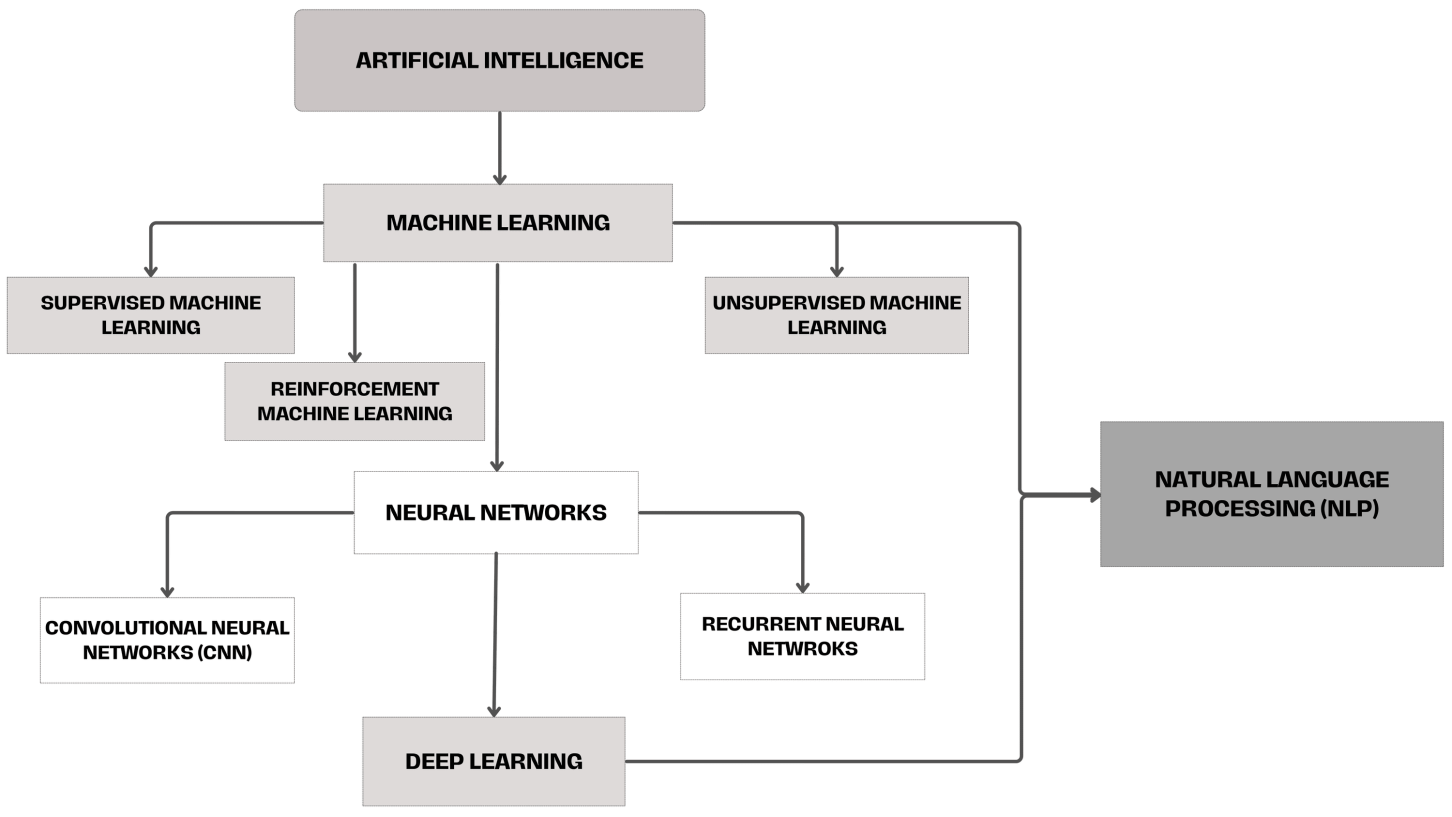
Flowchart representing subtypes of AI tools This flowchart outlines the hierarchical structure of AI subtypes, highlighting ML methods (supervised, unsupervised, and reinforcement learning), DL models such as CNNs and RNNs, and NLP as a key tool for language-based data. CNN, convolutional neural network; DL, deep learning; ML, machine learning; NLP, natural language processing; RNN, recurrent neural network Figure created by the authors using data from reference [39].

The classification of AI models is shown in Figure 3.

ML Models 

AI modalities vary in their readiness for HF applications. ML and DL are widely used, with ML excelling in pattern recognition and classification using various data types, including ECG, echocardiography, and EHRs. DL, particularly CNNs, is highly effective for image analysis, such as echocardiograms. CNN architectures such as ResNet and U-Net are proficient in data extraction from images, accurately detecting lesions and performing segmentation [39]. Long short-term memory (LSTM) networks show promise in AI-assisted health coaching and chatbots. In HF, LSTM networks can analyze longitudinal data from wearable devices and EHRs to predict possible disease deterioration and assist in selecting personalized treatment strategies.

Advanced ML techniques, such as gradient boosting machines (e.g., XGBoost) and support vector machines, have demonstrated strong predictive performance in complex clinical scenarios, including the prediction of HF readmissions [40]. Explainable AI (XAI) is crucial for trust and adoption, particularly in sensitive areas such as healthcare. Hybrid AI approaches that combine different modalities, for example, CNNs for imaging and large language models for EHR data, leverage the strengths of each method and can overcome individual limitations. Such hybrid frameworks show promise for comprehensive HF management, including early detection, risk stratification, and personalized treatment, with potential for expedited triage, timely therapy initiation, and improved patient outcomes [41,42].

Regulatory Frameworks 

The regulatory landscape for AI in healthcare emphasizes strict monitoring to ensure safety and efficacy. There is global recognition of the need to ensure that AI-driven devices and applications meet rigorous standards, particularly in the EU, UK, US, Japan, and South Korea. However, approaches differ. The EU has adopted a risk-based comprehensive framework through the AI Act, prioritizing assessment of high-risk AI systems [43]. In the US, the FDA focuses on SaMD, promoting transparency and enabling real-world performance monitoring. In the UK, the Medicines and Healthcare products Regulatory Agency (MHRA) and National Institute for Health and Care Excellence (NICE) advocate evidence-based evaluation, with attention to real-world applicability and seamless integration into existing clinical systems. In Japan and South Korea, the Ministry of Health, Labour and Welfare (MHLW) and Ministry of Food and Drug Safety (MFDS) prioritize conventional safety and efficacy systems [44-47].

Despite these differences, a common challenge persists: global frameworks currently lack clear guidance on algorithmic bias assessment. Variations in implementation strategies, specificity, and approaches to continuous learning highlight the evolving nature of regulatory systems in response to rapid AI advances in healthcare. Table 2 presents a comparative analysis of regulatory approaches for AI tools enforced by different countries.

**Table 2:** Comparative analysis of regulatory approaches for AI tools across different regions ML, machine learning; MHRA, Medicines and Healthcare products Regulatory Agency; NICE, National Institute for Health and Care Excellence; MHLW, Ministry of Health, Labour and Welfare; MFDS, Ministry of Food and Drug Safety; SaMD, software as a medical device Table created by the authors using data from references [43-47].

Feature	EU (AI Act)	UK (MHRA/NICE)	US (FDA)	Japan (MHLW)	South Korea (MFDS)
Approach	Risk-based, emphasizing high-risk AI systems [43]	Evidence-based, focusing on real-world evidence [46]	Risk-based, with a focus on SaMD [44]	Safety and efficacy focus [47]	Safety and efficacy focus [45]
Key focus	Risk assessment, transparency, accountability [43]	Real-world evidence, workflow integration [46]	Transparency, real-world performance monitoring, continuous learning [44]	Safety, efficacy, and data handling [47]	Safety, efficacy, and data handling [45]
Validation pathways	Lacks detailed clinical validation pathways [43]	Emphasis on Evidence Standards Framework for Digital Health Technologies; practical applicability concerns [46]	Draft guidance on AI/ML-based SaMD; challenges with continuous learning algorithms [44]	Focus on traditional safety and efficacy protocols [47]	Focus on traditional safety and efficacy protocols [45]
Algorithmic bias	General emphasis on risk mitigation; more detailed protocols needed [43]	Concerns regarding practical applicability of guidelines in real-world settings [46]	Emphasis on transparency but struggles with defining clear metrics for continuous learning algorithms [44]	Needs more detailed protocols for algorithmic bias assessment [47]	Needs more detailed protocols for algorithmic bias assessment [45]
Continuous learning	Challenges in adapting to continuous learning algorithms [43]	Challenges in adapting to continuous learning algorithms [46]	Struggles with defining clear metrics for continuous learning algorithms [44]	Challenges in adapting to continuous learning algorithms [47]	Challenges in adapting to continuous learning algorithms [45]

AI in HF Prediction and Risk Stratification

**Figure 4:**
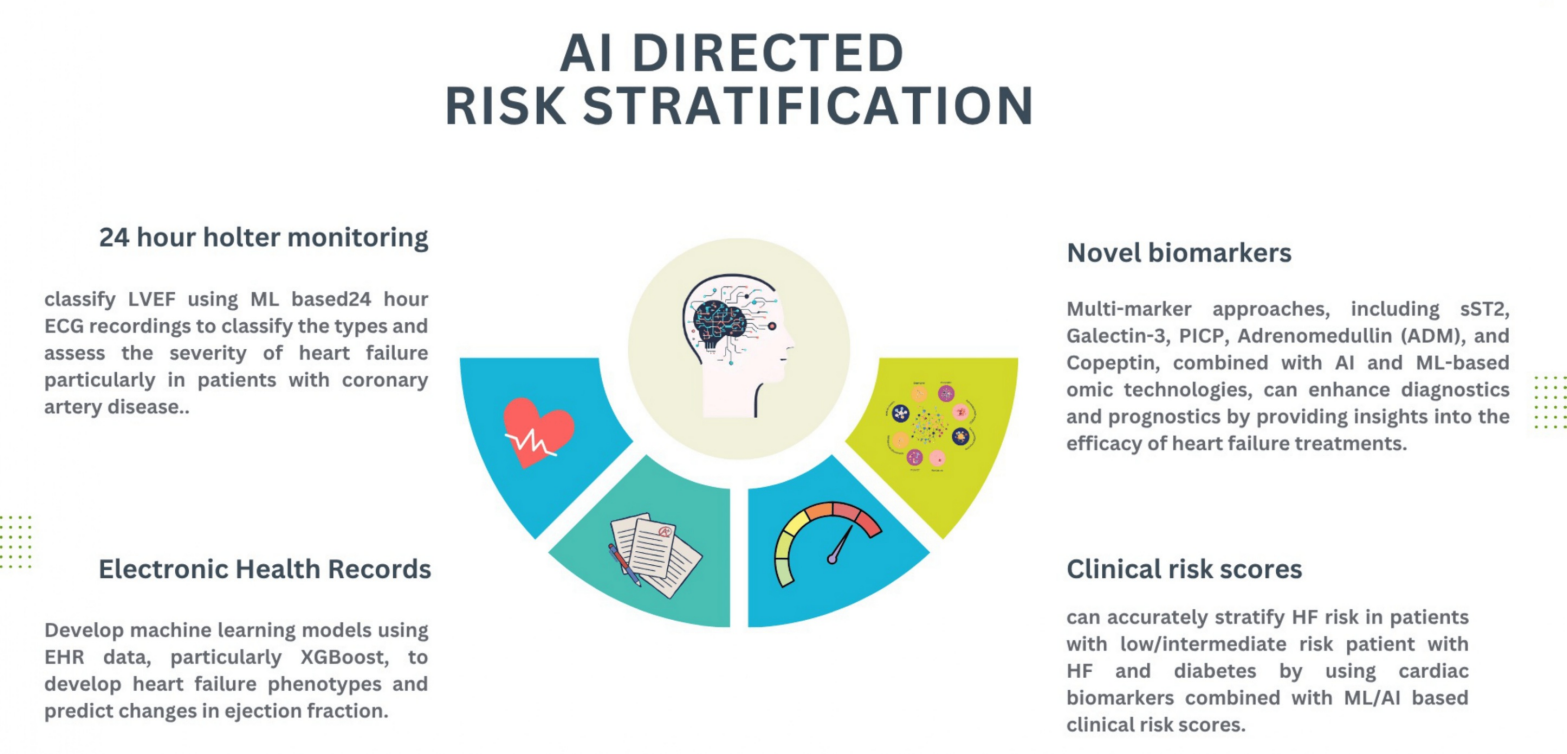
Different AI-driven models for enhanced risk stratification in HF The figure illustrates models integrated within existing diagnostic and clinical tools designed to improve risk stratification in HF, using advanced AI-based approaches to predict patient outcomes. ADM, adrenomedullin; EHR, electronic health records; HF, heart failure; LVEF, left ventricular ejection fraction; ML, machine learning; PICP, procollagen I carboxyterminal propeptide; SSTR2, somatostatin receptor 2; XGBoost, extreme gradient boosting Figure created by the authors using data from references [20,22,50].

The diagnosis of HF and the prediction of early mortality risk are areas where AI has demonstrated significant success (Figure 4). Pre-AI systems were less efficient in providing detailed insights, whereas AI-based predictive models offer substantial advantages by leveraging rich data from electronic medical records, including ECG and imaging studies. Traditional methods primarily aimed to stratify patients according to the complexity of their clinical presentation [48]. Identifying heart function deficiencies early is crucial, and DL models have shown superior performance in predicting alterations in cardiac function, such as changes in EF. This capability allows the timely generation of alarm data for proactive intervention [49]. A novel approach using deep neural network (DNN)-based mortality event classifiers has demonstrated high accuracy in forecasting and assessing prognosis in acute HF patients in the ICU. DNN models outperform single ML models and ensemble learning models, achieving superior accuracy, precision, recall, and area under the ROC curve [15].

AI in Diagnosis and Detection of HF

AI-driven ECG analysis has transformed HF diagnostics by detecting subtle waveform abnormalities, including QRS duration, fragmentation, and voltage variations, which correlate with HF progression [51]. AI-enhanced, ECG-enabled stethoscopes facilitate point-of-care screening, particularly for identifying LVEF ≤40%, a critical threshold for HF management [48]. A Mayo Clinic study involving over 45,000 patients demonstrated that an AI-powered ECG system achieved high specificity (86.3%) and sensitivity (93.0%) in detecting subclinical left ventricular dysfunction [52].

AI applications in echocardiography have markedly improved the identification of structural heart disease and diastolic dysfunction. DL models, such as EchoNet Dynamic, enable beat-to-beat cardiac function assessment, allowing early detection of ventricular dysfunction even before clinical symptoms appear [10]. ML algorithms applied to transthoracic echocardiography have been validated against expert sonographers, demonstrating 80% feasibility for use by non-specialists [53]. These advances hold promise for expanding HF diagnostics beyond specialized centers, improving early detection, and enabling timely intervention strategies.

NLP applied to EHRs has enhanced detection of undiagnosed HF with preserved EF (HFpEF), which is often overlooked in conventional clinical workflows [49]. NLP-driven AI systems analyze physician notes and structured clinical data, facilitating automated risk identification while simultaneously reducing physician workload [49].

Optimization of the Selection of Treatment and Provision of Personalized Care

AI models have demonstrated potential in identifying optimal HF therapies by predicting individualized patient responses. ML algorithms have classified patients based on their response to spironolactone, a key therapy for HFpEF, and the likelihood of treatment success. Results have shown a significant reduction in adverse outcomes among responders to this treatment [54]. In another study by Bayes-Genis et al., AI elucidated molecular mechanisms of sodium-glucose co-transporter 2 inhibitors, such as empagliflozin, providing deeper insight into their role in HF treatment [55].

AI models, including clustering algorithms and the random forest algorithm, have identified HF phenotypes most likely to benefit from cardiac resynchronization therapy, optimizing resource allocation and improving response rates [56]. These algorithms are particularly useful in borderline cases where traditional selection criteria may be insufficient, enabling more precise and patient-specific treatment selection.

AI has also been employed to predict adverse drug reactions, such as digoxin toxicity, using decision-tree algorithms and ML-based risk assessment [57]. These predictive tools enhance drug safety, reducing the risk of HF exacerbations linked to inappropriate prescriptions. ML-based clustering techniques have been further applied to predict and understand adverse events following left ventricular assist device (LVAD) implantation. Through clustering, sequential patterns and associations among post-implantation events were extracted, while artificial neural network-based image recognition algorithms quantified infection severity at LVAD driveline exit sites [58].

Remote Monitoring and AI-Driven Patient Management

Sensors in wearable medical devices (or “wearables”) can collect and process a variety of physiological data [59]. These devices can be worn on the wrist, attached to clothing, or affixed to the skin. Heart rate and rhythm can be monitored using optical sensors, such as photoplethysmography (PPG), often combined with accelerometers in activity monitors and smartwatches [59]. AI-driven wearable sensors facilitate continuous, real-time monitoring of HF patients by tracking heart rate variability, weight fluctuations, and arrhythmias, providing early warnings for clinical deterioration [60]. AI-based predictive analytics can proactively alert healthcare providers, ensuring timely intervention and preventing avoidable hospitalizations [60].

WHO defines mHealth as “medical and public health practice supported by mobile devices, such as mobile phones, patient monitoring devices, personal digital assistants, and other wireless devices” [61]. mHealth technologies have simplified remote patient monitoring and disease surveillance [61]. Studies primarily focus on diagnosing arrhythmias and monitoring conditions such as HF and myocardial infarction [61]. The combined use of the Internet of Things (IoT), AI, and ML has the potential to transform patient care and engagement [62]. IoT-based remote monitoring solutions enhance adherence to HF management protocols, and AI-integrated mHealth applications improve patient engagement by promoting daily weight tracking and medication compliance, thereby reducing the risk of HF exacerbations [62].

Barriers and Boundaries in AI Integration

While AI offers many benefits, several challenges must be considered in its integration into HF management. AI models are often trained on specific patient populations, which limits their generalizability across diverse demographics. Studies have highlighted racial and socioeconomic biases, emphasizing the need for multicenter validation and integration of diverse datasets to ensure equitable AI applications [63]. In addressing biases within HF models, Segar et al. demonstrated that including social determinants of health, such as education, housing quality, income, and other factors, within ML models improves calibration and reduces data disparities among these groups [64].

Most AI-based studies rely on retrospective data, which limits the establishment of causal relationships. Future research should include prospective studies with internal and external validation to support reliable and clinically applicable AI [64]. The accuracy of AI models heavily depends on the quality of input data. Inconsistent or inaccurate data from mHealth devices can lead to erroneous predictions, potentially compromising patient safety. Furthermore, many ML models developed for HF management lack external validation, which is essential before clinical implementation, as model performance may decline when applied to external datasets [65].

HF is a heterogeneous disorder with multidimensional variables, and excessive input features can degrade model performance in disease identification and classification. This can be mitigated by grouping variables into “super features,” preserving both performance and interpretability [65]. Conversely, models with limited features risk overfitting, reducing their validity [65].

The “black box” nature of AI models is a major barrier to clinical adoption. Improving explainability through frameworks such as SHapley Additive exPlanations (SHAP) and Local Interpretable Model-Agnostic Explanations (LIME) is critical for enhancing clinician trust and usability [66,67]. Human supervision further improves reliability and accountability and reduces bias. Physician expertise is often required for implementing and interpreting integrated models combining PPG and ECG, which may not be readily available in all healthcare settings [2]. Institutional training programs, such as the Mayo Clinic AI Academy, provide tiered education from foundational AI awareness to advanced clinical applications, helping address workforce training requirements.

Ethical considerations, including patient privacy, data security, and regulatory compliance, are also crucial. Adherence to the General Data Protection Regulation and FDA guidelines ensures data integrity and clinical safety [5]. Another barrier is funding; support from government agencies, nonprofit organizations, private foundations, and tech companies is essential for developing and implementing AI algorithms. For example, the National Health Service (NHS) AI Lab programs in the UK provided grants and procurement pilots, enabling funding and adoption of AI tools for imaging and triage pathways [68].

**Figure 5:**
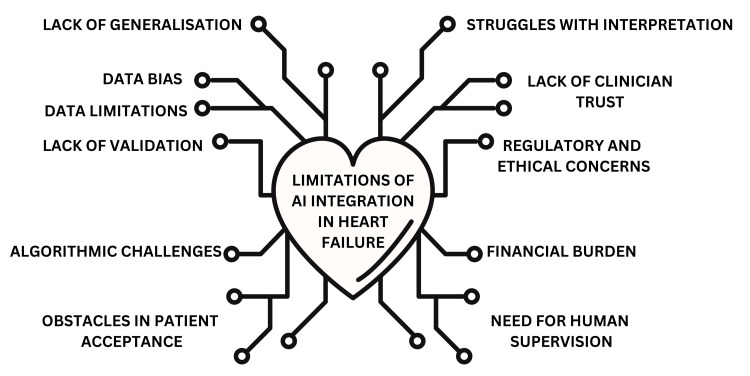
Challenges and limitations of AI integration in HF management This figure highlights key challenges in integrating AI into HF management. It illustrates technical, ethical, and clinical barriers, including data bias, lack of validation, financial constraints, and clinician distrust. These limitations affect AI’s reliability, acceptance, and effectiveness in real-world HF care. HF, heart failure Figure created by the authors using the data from references [2,5,63-67].

Figure 5 summarizes the challenges and limitations of utilizing AI in HF management.

Future Directions

The integration of AI into HF management is poised to redefine cardiovascular care, with ongoing advancements in predictive analytics, diagnostic imaging, and personalized treatment selection. One promising innovation is the development of a digital heart twin, which can theoretically generate ECG and acoustic signals to train ML models capable of automatically distinguishing individual heart sound components from real phonocardiograms. Such systems can enable early interventions to prevent hospital readmissions and adverse events. Digital twins can also provide real-time alerts upon detecting deterioration in a patient’s condition, allowing clinicians to respond swiftly and deliver timely care [69].

A notable digital twin model was developed through a partnership between the NHS in the UK and Imperial College London, focusing on patients with PAH. This model integrates data from wearables, imaging techniques, and other diagnostic tests to create a personalized simulation of a patient’s heart. The study aims to include a population of 100 patients [70]. Additional opportunities for future HF research include myocardial recovery assessment for treatment response prediction, risk stratification, phenogrouping, and the integration of multi-omic datasets [29].

In terms of regulatory guidance, the FDA is adopting a new approach that accounts for the evolving nature of AI/ML models and software, classifying them as SaMD. This includes the Innovative Science and Technology Approaches for New Drugs (ISTAND) pilot program, which explores AI/ML software as a potential Drug Development Tool (DDT) [44]. The EU AI Act is also expected to publish the AI Tools framework in the second quarter of 2025 [43].

**Figure 6:**
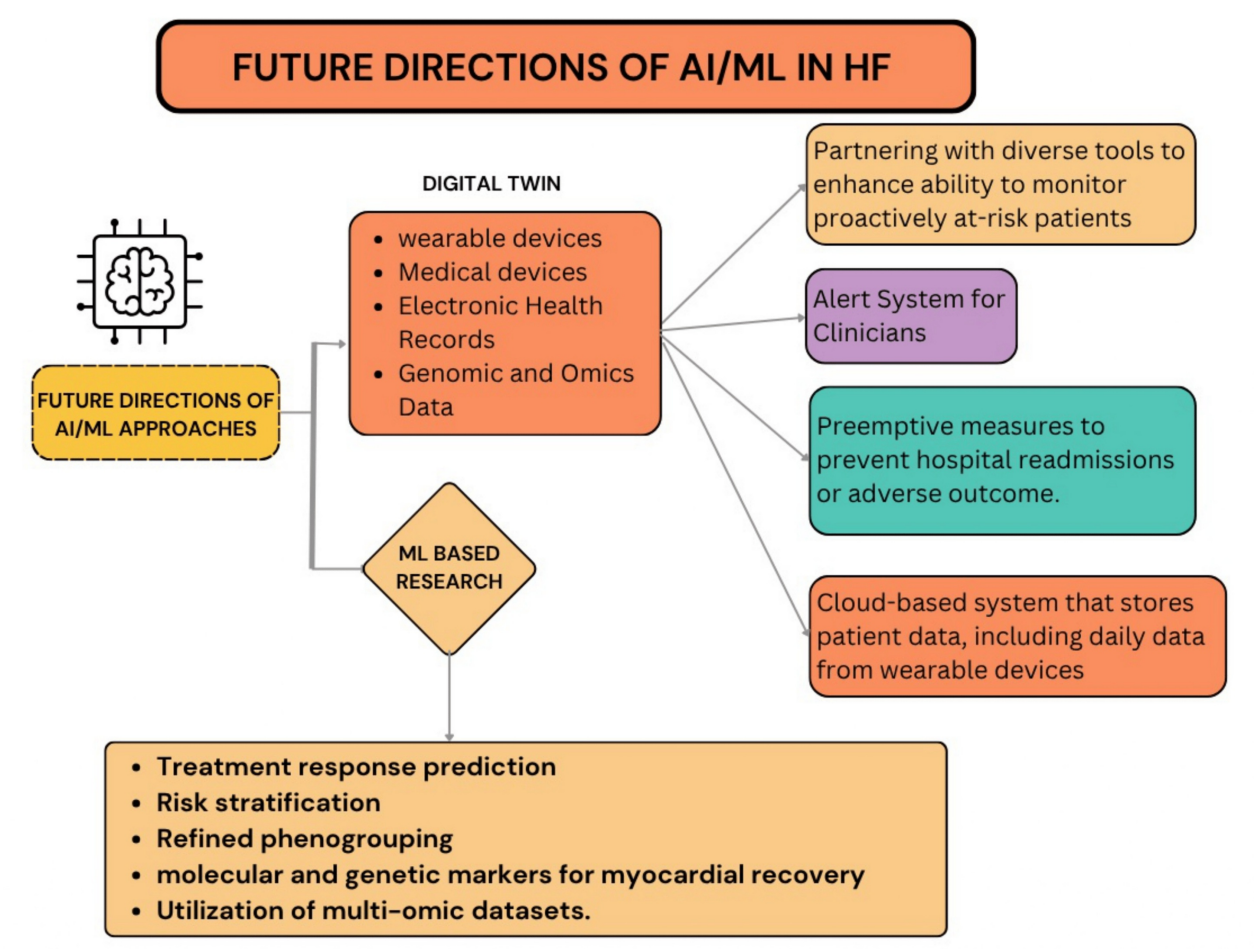
Promising advancements and research opportunities in HF management This figure highlights emerging advancements and research opportunities in HF management using AI and ML, showcasing novel technologies, future research directions, and potential improvements in patient care. HF, heart failure; ML, machine learning Figure created by the authors using the data from references [69,71].

Although challenges remain, particularly regarding generalization, interpretability, and regulatory compliance, the potential benefits of AI in HF care are substantial (Figure 6). By bridging AI-driven insights with real-world clinical application, AI has the potential to shift HF management from reactive care to proactive, precision-medicine-based strategies, ultimately reducing mortality, improving quality of life, and enhancing patient outcomes.

## Conclusions

AI has demonstrated significant potential in improving HF management through risk prediction, early diagnosis, treatment optimization, and remote monitoring. ML and DL models have enhanced risk stratification and prognostication, providing more precise assessments than traditional clinical methods. AI-driven tools, including ECG and echocardiography-based diagnostics, EHR integration, and predictive analytics, contribute to earlier detection and more targeted interventions. Despite these advancements, challenges such as algorithmic bias, model interpretability, regulatory constraints, and data security concerns must be addressed to enable reliable clinical integration. Ensuring multicenter validation, ethical oversight, and clinician engagement will be crucial to support the responsible adoption of AI in HF care.

A definitive assessment of AI models in HF requires prospective clinical trials conducted in real-world populations with diverse patient groups to ensure external validity. Telemonitoring provides a means to validate AI tools in practice, allowing real-time tracking of heart rate variability, weight fluctuations, and arrhythmias, with alerts sent to clinicians when early signs of clinical deterioration are detected. Future developments, including digital twins and multi-omics-driven models, may further refine personalized treatment approaches, shifting HF management toward proactive, data-driven strategies. Additionally, establishing standardized governance frameworks aligned with regulatory authorities such as the FDA and MHRA is essential to ensure the safe, effective, and equitable deployment of AI models in HF care. With continued research and structured implementation, AI has the potential to enhance clinical decision-making, improve patient outcomes, and contribute to more effective HF management.
